# Physico-chemical properties of biodiesel manufactured from waste frying oil using domestic adsorbents

**DOI:** 10.1088/1468-6996/16/3/034602

**Published:** 2015-05-05

**Authors:** Samir Abd-elmonem A Ismail, Rehab Farouk M Ali

**Affiliations:** Department of Biochemistry, Faculty of Agriculture, Cairo University, 12613, Giza, Egypt

**Keywords:** adsorbents, filter, transesterification, biodiesel characteristics, waste frying oil, COX value

## Abstract

We have evaluated the efficiency of sugar cane bagasse ash (SCBA), date palm seed carbon (DPSC), and rice husk ash (RHA) as natural adsorbents and compared them with the synthetic adsorbent Magnesol XL for improving the quality of waste frying oil (WFO) and for the impact on the physicochemical properties of the obtained biodiesel. We measured moisture content, refractive index (RI), density, acid value (AV), iodine value (IV), peroxide value (PV), and saponification value (SV), as well as fatty acid profile. Purification treatments with various levels of adsorbents caused significant (P ≤ 0.05) decreases in free fatty acids (FFAs), PVs, and IVs. The highest yields (86.45 and 87.80%) were observed for biodiesel samples produced from WFO treated with 2% Magnesol and 3% of RHA, respectively, followed by samples treated with 2 and 3% of DPSC or RHA. Pre-treatments caused a significant decrease in the content of C 18:2 linoleic acids, consistent with a significant increase in the content of monounsaturated and saturated fatty acids (MUFA) in the treated samples. The highest oxidation value (COX) (1.30) was observed for biodiesel samples produced from WFO without purification treatments. However, the lowest values (0.44–0.73) were observed for biodiesel samples produced from WFO treated with different levels of adsorbents. Our results indicate that pre-treatments with different levels of adsorbents regenerated the quality of WFO and improved the quality of the obtained biodiesel.

## 1. Introduction

Biodiesel plays a major role in the energy sector due to its similar combustion properties with petroleum. Furthermore, biodiesel is sometimes superior to petroleum diesel with improved physical and chemical properties, such as a higher flash point, higher cetane number, ultralow sulfur content, better lubricity, improved biodegradability, and a smaller carbon footprint [1–3]. Biodiesel, an alternative diesel fuel, is made from renewable biological sources such as vegetable oils and animal fats [4]. In accordance with the US Standard Specification for Biodiesel (ASTM 6751), biodiesel is defined as a fuel comprised of mono-alkyl esters of long-chain fatty acids derived from vegetable oils or animal fats [5]. It has similar physicochemical properties to conventional fossil fuel and can, consequently, entirely or partially substitute for fossil diesel fuel in compression ignition engines [6].

**Figure 1.**
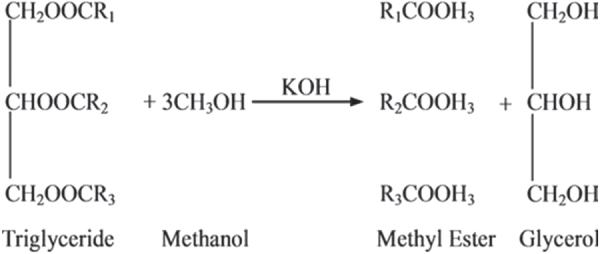
The transesterification of vegetable oil or fat with methanol alcohol.

Biodiesel, which is accepted as an attractive alternative fuel, is prepared by transesterification of vegetable oils and animal fats with an alcohol in the presence of a catalyst (figure 1). However, the land use for production of edible oil for biodiesel feedstock competes with the use of land for food production. Moreover, the price of edible plant and vegetable oils is usually higher than petrodiesel. The use of waste cooking oil as biodiesel feedstock reduces the cost of biodiesel production [7, 8]. A huge amount of waste cooking oil generated from the restaurant and food process industries is disposed without prior treatment. The Energy Information Administration estimated that 100 million gallons of waste cooking oil is produced per day in the USA [9], while UK and EU countries generate approximately 200 000 tons and 700 000–1 000 000 tons of waste cooking oil in a year, respectively [8]. The wastes can be used if they are purified; used frying oil is purified by removing its free fatty acids (FFAs) by magnesium oxide adsorption [10]. In this respect, there are various methods that can be used to remove these products, such as filtration through special membranes [11, 12] and by using different adsorbent materials, including both natural and synthetic adsorbents. Usually, active forms of carbon, calcium, silica, aluminum, and magnesium are major constituents of such products [13–17]. Many reports have appeared on the production of low-cost adsorbents using cheaper and readily available materials [17–19].

The biodiesel fuel product should satisfy the registration requirements for fuels and fuel additives established by the Unites States Environmental Protection Agency (EPA) under section 211 of the Clean Air Act and the requirements of the American Society of Testing and Materials (ASTM) D6751 [5]. The aim of the present investigation was evaluation of the efficiency of sugar cane bagasse ash (SCBA), date palm seed carbon (DPSC), and rice husk ash (RHA) as natural adsorbents in comparison with Magnesol XL as a synthetic adsorbent in improving the quality of waste frying oil (WFO), as well as their impact on the physicochemical characteristics of the obtained biodiesel.

## 2. Materials and methods

### 2.1. Materials

WFO (water content 0.29%, acid value (AV) 1.55 (mg KOH g^−1^ Oil), peroxide value (PV) 14.84 (milliequivalent O_2_ kg^−1^), and iodine value (IV) 62.41 g (I_2_/100 g oil)) was collected from Food Industries Company, Sadat City, Menufia, Egypt. Sugar cane bagasse (SCB) was obtained from the Hawamdiah sugar refinery company, El-Hawamdiah, Egypt. Date palm seeds were collected from the jam industries of Egypt. Rice husk was also obtained from a farm in Menufia, Egypt. The adsorbent Magnesol XL (a hydrous, white, amorphous, and odorless synthetic magnesium silicate) was obtained from the Magnesol Product Division, Reagent Chemical and Research, Inc., in Houston, TX, USA. The other chemicals used in the current study were obtained from the Sigma Chemical Company (St. Louis, MO, USA).

#### 2.1.1. Preparation of adsorbents

##### 2.1.1.1. Preparation of sugar cane bagasse ash (SCBA)

SCB was prepared according to the method described by Ali and El Anany [17]. It was first washed thoroughly with distilled water to remove the dust particles and then dried in an oven at 100 °C for 72 h. The dried product was ground in a grinder (Perten, USA) to a particle size of about 190 nm, placed in a crucible, carbonized in air in a muffle furnace (Furnace 5000; Thermolyne, IA, USA) at 650 °C for 12 h, and then cooled to room temperature in a desiccator and stored in glass screw-capped bottles.

##### 2.1.1.2. Carbonization of date palm seeds

Date palm seed carbon (DPSC) was prepared according the method described by El Nemr *et al* [19]. Date palm seeds were washed with tap water, cut into small pieces, and dried at 150 °C for 48 h. The dried product was treated with concentrated sulfuric acid (H_2_SO_4_ 98%, Sp.gr.1.84) at a ratio of 2:1 (v/v). The obtained black product was kept in an oven at 200 °C for 10 h. The carbonized material was washed with distilled water to remove the free acids and then dried at 105 °C. The carbon obtained was ground and sieved to a particle size ≤0.150 mesh and kept in a glass bottle until use.

##### 2.1.1.3. Rice husk ash (RHA)

RHA was prepared according the method of AOAC [20]. The husk was ground in a grinder (Perten, USA) to a particle size of about 180 nm, placed in a crucible, carbonized in air in a muffle furnace (Furnace 5000; Thermolyon, IA, USA) at 550 °C for 12 h, and then cooled to room temperature in a desiccator and stored in glass screw-capped bottles.

#### 2.1.2. Pre-treatment of used waste frying oil (WFO)

The adsorbents, i.e., SCBA, DPSC, and RHA, were mixed individually with 2000 ml of WFO at levels 1, 2, and 3% (w/v) and Magnesol XL 2% (w/v), then mechanically stirred for 60 min at 105 °C. The slurry was vacuum filtered through a Whatman No. 41 filter paper (Whatman International Ltd, Maidstone, UK). Vacuum filtration facilitated the flow of oil through the filter paper. The unpurified WFO was vacuum filtered through a Whatman No. 41 filter paper and used for producing the control samples.

#### 2.1.3. Biodiesel production

Five hundred grams of treated and untreated WFO were added individually into a 1000 ml round-bottom flask equipped with a condenser. After the oil was heated to 65 °C, the solution of sodium hydroxide (5.0 g) in methanol (144.82 ml, 6:1 molar ratio of methanol to oil) was slowly added into the reaction. The reaction temperature of the transesterification process was set at 60 °C in order to prevent the vaporization of the methanol from the reacting mixture during the biodiesel production process. The reaction was allowed to proceed for 110 min. At the end of the incubation, the mixture was transferred into a reparatory funnel, left for 24 h, and then the biodiesel was separated from the glycerol. The biodiesel was then washed with hot deionized water (50 °C) five times to remove the glycerol, catalyst, and other impurities [21].

### 2.2. Methods

#### 2.2.1. Analytical methods

These tests were conducted on oil and biodiesel samples.

##### 2.2.1.1. Moisture content

The moisture content was determined according to the AOAC official method [20].

##### 2.2.1.2. Acid value (AV)

The AV was determined according the AOAC method (969.17) [20] as follows. A known weight (2 g) of the oil or biodiesel sample was dissolved in a neutral ethyl alcohol (30 mL); the mixture was boiled in a water bath for 2 min and then titrated with a potassium hydroxide solution (0.1 N) in the presence of phenolphthalein as an indicator. Acid value is expressed as mg KOH required to neutralize the acidity in one gram of oil.

##### 2.2.1.3. Peroxide value (PV)

The PV was determined according to the AOAC method (965.33) [20]. A known weight of the oil or biodiesel sample (2.5 g) was dissolved in a mixture consisting of glacial acetic acid: chloroform (30 ml, 3:2, and v/v), then freshly prepared saturated potassium iodide solution (1 ml) was added. Distilled water (30 ml) was added then titrated slowly with sodium thiosulfate solution (0.1 N) in the presence of a starch solution (1%) as an indicator. PV is expressed as the milliequivalent of the O_2_ kg^−1^ sample.

##### 2.2.1.4. Iodine value (IV)

The IV was determined using the Hanus method, as described in AOAC (920.158) [20]. A known weight of the oil or biodiesel sample (0.2 g) was dissolved in chloroform (20 ml), then Hanus iodine (I_2_ + Br/ACOH) solution (25 ml) was added and left in the dark for 30 min. Potassium iodide solution (10 ml, 15%) was added, followed by freshly distilled water (100 ml), and the excess iodine was titrated by sodium thiosulfate (0.1 N) until the yellow color of the solution had almost disappeared. Titration was continued after adding a few drops of starch as an indicator until the blue color had entirely disappeared. A blank was conducted where the total halogen content of the Hanus solution (25 ml) was determined by sodium thiosulfate solution without the addition of oil. IV is expressed as grams of I_2_ absorbed by 100 g oil or biodiesel sample.

##### 2.2.1.5. Saponification number

Saponification value (SV) was determined according to the AOAC method (920.160) [20]. A known weight of the oil or biodiesel sample (5 g) was heated with alcoholic potassium hydroxide (50 ml, 0.5%) for about 30 min. The reaction mixture was cooled down and then titrated with hydrochloric acid solution (0.5 N) using phenolphthalein as an indicator. A blank was performed where the same volume of alcoholic potassium hydroxide solution without oil was treated similarly as in the experiment. The saponification number is expressed as mg KOH required to saponify 1 g oil or biodiesel sample.

##### 2.2.1.6. Refractive index (RI)

The refractive index of the oil or biodiesel sample was determined according to the AOAC method (977.17) [20] using an Abbe refractometer (NYRL -3—Leica Mark, Leica Inc., Buffalo, New York).

##### 2.2.1.7. Density

The density of the samples was determined by a mass over volume measurement at 40 °C.

##### 2.2.1.8. Yield measurement

According to Leung and Guo [22], the yield of the product was calculated by using the following equation

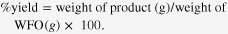


##### 2.2.1.9. Oxidation value (COX)

The calculated oxidation stability value (COX) of the samples was calculated by applying the formula proposed by Fatemi and Hammond [23] as follows:

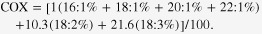


##### 2.2.1.10. Fatty acid composition

Capillary gas chromatography (HP 6890) was used for the qualitative and quantitative determinations of the fatty acid methyl esters (FAMEs) of the samples and reported in relative area percentages. The FAMEs were identified using a gas chromatograph equipped with a DB-23 capillary column (60 m, 0.32 mm ID, 0.25 *μ*m film thickness) and a flame ionization detector. The nitrogen flow rate was 3 mL min^−1^; hydrogen and airflow rates were 40 and 450 mL min^−1^, respectively. The oven temperature was programmed from 150–170 °C at a rate of 10 min, then raised to 192 °C at a rate of 5 °C min^−1^, kept at this temperature for 5 min, and then raised again to 220 °C at a rate of 10 °C min^−1^and kept at this temperature for 3 min. The injector and the detector temperatures were 230 °C and 250 °C, respectively. FAMEs were identified by comparing their retention times with a known fatty acid standard mixture. Peak areas were automatically computed by an integrator.

#### 2.2.2. Statistical analysis

Data are expressed as mean ± standard deviation (SD) of three replicates. The data were analyzed by analysis of variance according to the procedures outlined by Gomez and Gomez [24]. Duncan’s multiple-range test was used to determine the differences among samples. Significant levels were defined as probabilities of 0.05 or less.

## 3. Results and discussions

To evaluate the efficiency of the adsorbents under investigation in the removal of the oxidized products and their impact on the physicochemical properties of the obtained biodiesel, some physicochemical properties were measured: moisture content, RI, density, AV, PV, SV, and IV. Oxidation products, which may include gums, acids, and peroxides, aredetrimental to the performance of diesel engines [25].

### 3.1. Moisture content

The moisture content of biodiesel from WFO as well as WFO treated with different adsorbents was not detected, which reflects the quality of the obtained biodiesel. The maximum amount of allowable water content in biodiesel as specified in ASTM standard D6751 [5] is 0.050% vol [5]. High moisture content in biodiesel may cause many problems such as water accumulation and microbial growth in fuel handling, storage, and transportation equipment [26]; it is often caused by improper treatment after processing [26] or by the absorption of atmospheric moisture during storage.

### 3.2. Refractive index (RI)

**Figure 2.**
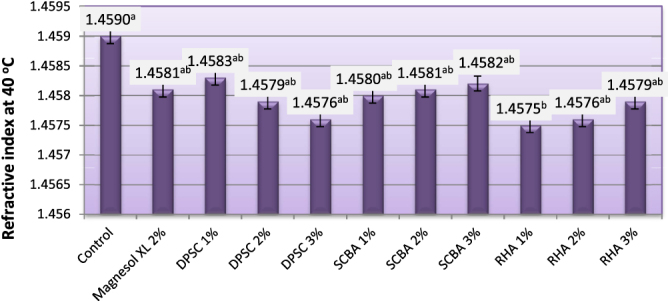
Refractive index at 40 °C of biodiesel produced from WFO and WFO treated with various levels of adsorbents. Values are means of three determinations. Values followed by the same letter (^a, b, c, d, e^) are not significantly different (*p* < 0.05) by Duncan’s multiple range test. Least significant difference (LSD) at 5% level = 0.0008.

The RI is a parameter that relates to molecular weight, fatty acid chain length, degree of unsaturation, and degree of conjugation. The response of material to light beams depends on real and imaginary parts of the RI, and the absorption coefficient is related to the imaginary part. Moreover, when the biodiesel temperature is near the cloud point, a cloudy state appears, and the RI changes; hence, it is a significant parameter to evaluate the state of a biodiesel. In addition, the RI also depends on the concentration of saturated and unsaturated fatty acids. Thus, it is a useful parameter for standardization of the product [27]. The RIs of biodiesel produced from WFO and WFO treated with different levels of adsorbents are shown in figure 2. RI values of the obtained biodiesel ranged from 1.4575–1.4590. These results are in good agreement with those of Domínguez *et al* [28] and Ullah *et al* [29], who reported that pure biodiesel possesses an RI in the range of 1.45. Biodiesel produced from WFO without treatments had significantly (*p* ≤ 0.05) the highest RI value of 1.4590 at 40 °C. The purification process using different levels of Magnesol XL, SCBA, DPSC, and RHA caused insignificant (*p* ≤ 0.05) decreases in the values of the RI. However, the purification process with 1% of RHA caused significant decreases in RIs. The reductions in the RI due to treatment with various adsorbents indicate the removal of some unsaturated compounds resulting from the frying process. The highest efficiency for removing some unsaturated compounds was observed for biodiesel samples produced from WFO treated with 1% of RHA. No significant differences were observed between the efficiency of Magnesol XL 2%; 1, 2, and 3% DPSC; or SCBA; as well as 2 and 3% of RHA on lowering the RI of treated WFO.

### 3.3. Density

**Figure 3.**
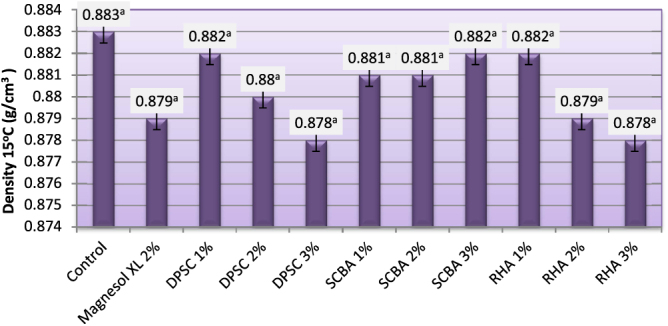
Density of biodiesel produced from WFO and WFO treated with various levels of adsorbents. Values are means of three determinations. Values followed by the same letter (^a, b, c, d, e^) are not significantly different (*p* < 0.05) by Duncan’s multiple range test. Least significant difference (LSD) at 5% level = 0.116.

Density is an important fuel property, because injection systems, pumps, and injectors must deliver an amount of fuel precisely adjusted to provide proper combustion [30]. The density values must be maintained within tolerable limits to allow optimal air-to-fuel ratios for complete combustion. High-density biodiesel or its blend can lead to incomplete combustion and particulate matter emissions [31]. The default value of 40 °C density specified in ASTM D6751 [5] is 0.82–0.90 g cm^−3^. The density of the obtained biodiesel varied from 0.878–0.883 g cm^−3^. Density depends upon the raw materials used for biodiesel fuel production and the biodiesel methyl ester profile [32]. No significant differences in density were observed between biodiesels obtained from untreated frying oil and those obtained from frying oil treated with different adsorbents. The results from figure 3 show that the density values of both biodiesels obtained from purified and unpurified oils meet the density value specified by the European Standard EN 14214 of biodiesel [33].

### 3.4. Acid value (AV)

**Figure 4.**
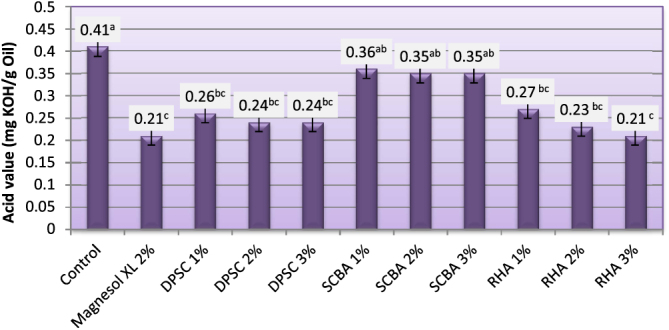
Acid value of biodiesel produced from WFO and WFO treated with various levels of adsorbents. Values are means of three determinations. Values followed by the same letter (^a, b, c, d, e^) are not significantly different (*p* < 0.05) by Duncan’s multiple range test. Least significant difference (LSD) at 5% level = 0.081.

Determination of AV is an important test to assess the quality of a particular biodiesel. The AV of the starting material can play an important role on the %FAME of the final product [34]. The maximum level of AV for pure biodiesel, as specified in ASTM standard D6751 [5], is 0.8 mg KOH g^–1^ [5]. The AV (mg KOH g^–1^ oil (of biodiesel produced from WFO and WFO treated with different levels of adsorbents are shown in figure 4. The AV of the obtained biodiesel varied from 0.21–0.41. Biodiesel produced from WFO without purification treatments had the highest acid value at 0.41. When fats, oils, and biodiesel go bad, there are some measurable effects. The acid number (ASTM D-664) [35] increases as the fatty acid breaks down into shorter chain acids. The results demonstrated that the purification treatment of WFO with different levels of adsorbents induced a significant lowering effect on the FFA levels. Biodiesel samples produced from WFO treated with different levels of adsorbent had lower levels of acidity, ranging from 0.21–0.36 mg KOH g^–1^ oil. These values were significantly (*p* ≤ 0.05) lower than the maximum value approved by both EN 14214 [33] and ASTM D6751 [5] standard fuels. In this respect, Ali and El-Anany [17] found that the acidity of used frying sunflower oil was remarkably reduced by treatment with different levels of SCBA and Magnesol XL. The FFA values after treatment dropped by 50–75%. The highest reductions of FFA content were observed in biodiesel samples produced from WFO treated with 2% Magnesol XL and 3% of RHA. No significant (*p* ≥ 0.05) differences were observed between the FFA content of biodiesel samples produced from WFO treated with 1, 2, and 3% of DPSC and those treated with 1 and 2% of RHA. The lowest reduction of FFA content was observed for biodiesel samples produced from WFO treated with SCBA. The high FFA content (>1%; w:w) will cause soap formation, and the separation of products will be exceedingly difficult; as a result, it has a low yield of biodiesel product [36]. In this regard, Felizardo *et al* [37] investigated the optimal conditions for biodiesel production from WFOs using sodium hydroxide as a catalyst. They advised that a pretreatment step would be required in order to obtain a higher percentage of fatty acid methyl ester.

### 3.5. Peroxide value (PV)

**Figure 5.**
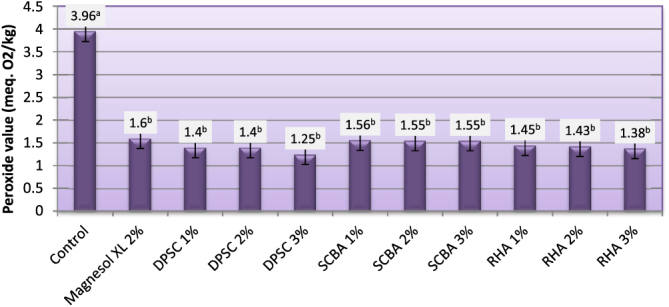
Peroxide value of biodiesel produced from WFO and WFO treated with various levels of adsorbents. Values are means of three determinations. Values followed by the same letter (^a, b, c, d, e^) are not significantly different (*p* < 0.05) by Duncan’s multiple range test. Least significant difference (LSD) at 5% level = 0.325.

The PV determination measures the presence of oxidative moieties (i.e., the portion of a molecule bearing characteristic oxidative properties) in a sample. The oxidative moieties usually found in biodiesel are hydroperoxides formed when oxygen from the air reacts with fatty esters. This usually is the first step in the oxidative degradation pathway of biodiesel [38]. The PVs of biodiesel produced from WFO and WFO treated with different levels of adsorbents are shown in figure 5. The PVs of the obtained biodiesel varied from 1.25–3.96 meq. O_2_ kg^–1^. The highest PV (3.96 meq. O_2_ kg^–1^) was recorded for biodiesel samples manufactured from WFO without purification treatments. Although the PV is not regulated by US and European standards, Dunn [39] reported that the increase in this value in biodiesel increases the cetane number, reducing the ignition time (the time between fuel injection in the cylinder and the start of ignition). The purification process with various levels of adsorbent caused significant (*p* ≤ 0.05) decreases in PVs. The adsorbents under investigation at different levels had the same efficiency in removing the hydroperoxides. The results indicate that biodiesel samples produced from WFO treated with different levels of adsorbent had the lower levels of hydroperoxides, ranging from 1.25–1.60 meq. O_2_ kg^–1^. The efficiency of various adsorbents in removing the peroxides was in decreasing order: DPSC > RHA > SCBA > Magnesol XL. Generally speaking, the adsorbents of the current investigation can be effectively used to lower the PVs of WFOs. These findings indicate that the biodiesels produced from WFO treated with different levels of adsorbents are less prone to oxidative rancidity. Oxidation results in the formation of hydroperoxide molecules, creating acids that attack metals, rubbers, and elastomers. Another unpleasant consequence of oxidation is that it also triggers polymerization processes–gum formation and sediments that tend to clog filters and nozzle injectors [40].

### 3.6. Saponification value (SV)

**Figure 6.**
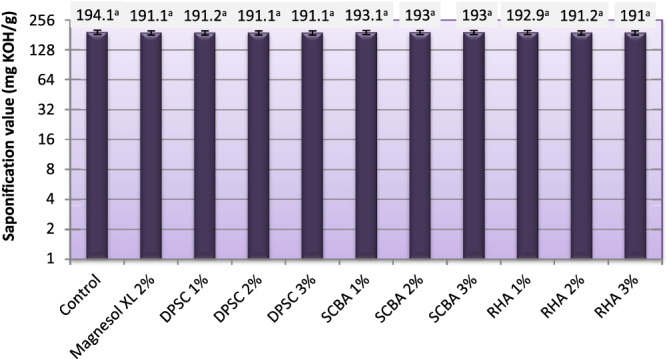
Saponification value of biodiesel of produced from WFO and WFO treated with various levels of adsorbents. Values are means of three determinations. Values followed by the same letter (^a, b, c, d, e^) are not significantly different (*p* < 0.05) by Duncan’s multiple range test. Least significant difference (LSD) at 5% level = 3.04.

The SV indicates the amount of saponifiable units (acyl groups) per unit weight of oil. A high SV indicates a higher proportion of low molecular weight fatty acids in the oil or vice versa [41]. The SV is used for measuring the average molecular weight of oil and expressed in milligrams of potassium hydroxide (mg KOH g^–1^ oil). The SVs of biodiesel produced from WFO and WFO treated with different levels of adsorbents are shown in figure 6. The SV of the obtained biodiesel varied from 191.03–194.10 mg KOH g^−1^. No statistically significant (*p* ≥ 0.05) differences in the SVs were found between biodiesel samples produced from WFO without purification treatments and those WFOs treated with different levels of adsorbents. The SV is related to the average molecular weight of the sample, but the acids that are present in glycerides or in methyl esters are the same. Only the change of glycerol by methanol is produced. In consequence, the average molecular weight does not change significantly, and so changes in the saponification value may not be observed [42].

### 3.7. Iodine value (IV)

**Figure 7.**
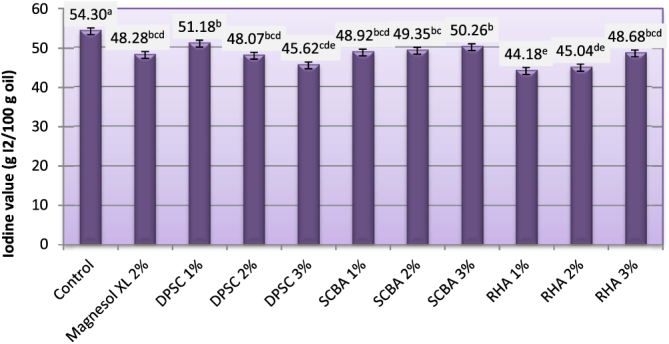
Iodine value of biodiesel produced from WFO and WFO treated with various levels of adsorbents. Values are means of three determinations. Values followed by the same letter (^a, b, c, d, e^) are not significantly different (*p* < 0.05) by Duncan’s multiple range test. Least significant difference (LSD) at 5% level = 2.67.

The IV is the measure of degree of unsaturation in oil. It is constant for a particular oil or fat. The IV is a useful parameter in studying oxidative rancidity and chemical stability properties of different oil and biodiesel fuels. A higher quantity of double bonds in the sample has greater potential to polymerize and, hence, lesser stability. The IVs of biodiesel produced from WFO and WFO treated with different levels of adsorbents are shown in figure 7. Oils with an IV above 125 are classified as drying oils; those with an IV of 110–140 are classified as semidrying oils. Those with IVs less than 110 are considered as nondrying oils. The IVs of the obtained biodiesel ranged from 44.18–54.30 g I2/100 g oil. Purification treatments with various levels of adsorbent caused significant (*p* ≤ 0.05) decreases in IVs. The results indicate that biodiesel samples produced from WFO treated with different levels of adsorbent had the lower IVs, ranging from 44.18–51.18. These values are significantly (*p* ≤ 0.05) lower than the maximum value (120 I2/100 g) approved by both EN 14214 [33] and ASTM D6751[5] standard fuels. IVs are useful for determining the overall degree of saturation of the oil, which is important for viscosity and cloud points. The lower the IV, the better the fuel will be as a biodiesel. Biodiesel from vegetable oils with high amounts of saturates (which means low IVs) will have a higher cetane number (CN), while biodiesel from vegetable oils with high amounts of unsaturates (high IVs) will have a lower CN. Unsaturation in the fatty acid chain is the most significant cause of lower CNs. IVs greater than 50 may result in decreased engine life but give better viscosity characteristics in cooler conditions [43].

### 3.8. Yield of biodiesel (%)

**Figure 8.**
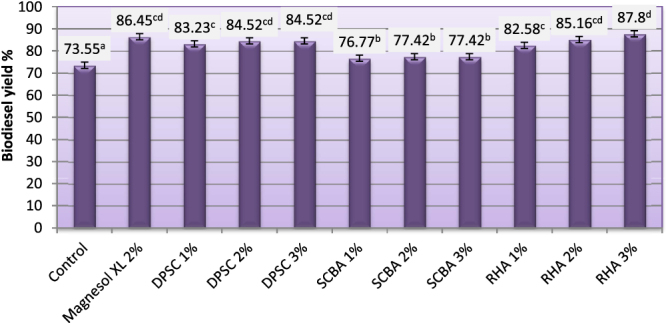
Yield of biodiesel produced from WFO and WFO treated with various levels of adsorbents. Values are means of three determinations. Values followed by the same letter (^a, b, c, d, e^) are not significantly different (*p* < 0.05) by Duncan’s multiple range test. Least significant difference (LSD) at 5% level = 2.68.

Biodiesel yield (%) for biodiesel samples produced from WFO and WFO treated with different levels of adsorbents are shown in figure 8. Biodiesel yield (%) of the obtained biodiesel ranged from 73.55–86.45%. Purification treatments with various levels of adsorbent caused significant (*p* ≤ 0.05) increases in the biodiesel yields. The highest yields (86.45 and 87.80%) were observed for biodiesel samples produced from WFO treated with 2% Magnesol and 3% of RHA, respectively, followed by samples treated with 2 and 3% of DPSC or RHA. However, the lowest yield (73.55%) was observed for control samples, which were produced from WFO without purification treatments. When the efficiency of esterification is relatively low, residual FFA produces soaps due to alkali catalysis, making the separation of biodiesel and alcohol difficult and simultaneously decreasing the final yield of biodiesel. WFO transesterification catalyzed by alkali only becomes possible when the AV of oil is less than 1 mg KOH g^–1^ oil. Higher percentages of FFA in the oil reduce the yield of the transesterification process. In fact, for oil with high FFA levels, the first esterification cannot meet the alkali catalyzed reaction conditions, which requires a second esterification in order to reduce acid value to less than 2 mg KOH g^–1^ oil [44].

### 3.9. Fatty acid compositions of the biodiesel produced from WFO and WFO treated with different levels of adsorbents

The properties of the triglyceride and the biodiesel fuel are determined by the amounts of each fatty acid that are present in the molecules. Chain length and number of double bonds determine the physical characteristics of both fatty acids and triglycerides [45]. Transesterification does not alter the fatty acid composition of the feedstock, and this composition plays an important role in some critical parameters of the biodiesel such as CN and cold flow properties [46]. The fatty acid compositions of the biodiesel produced from WFO and WFO treated with different levels of adsorbents are summarized in table 1. There are three main types of fatty acids that can be present in a triglyceride that is saturated (Cn:0), monounsaturated (Cn:1), or polyunsaturated with two or three double bonds (Cn:2,3). The most prominent fatty acids in the produced biodiesel were C 16:0 palmitic acid (43.51–50.72%) and C 18:1 oleic (cis-9-octadecenoic) acid (40.41–50.10%). A low level of C18:2 n-6 (8.77%) was found in biodiesel samples produced from WFO without purification treatments. Pre-treatments (purification process with various levels of adsorbents) caused a marked decrease in the content of C 18:2 linoleic acids, consistent with a marked increase in the content of monounsaturated and saturated fatty acids (MUFA) in the treated samples. The COS value, which is based on unsaturated fatty acid percentages present in the samples, is a beneficial element usually taken as an evaluation of the oil’s tendency to undergo auto-oxidation [23]. The results of the COX values for the biodiesels under study are shown in table 1. The highest COX value (1.30) was observed for biodiesel samples produced from WFO without purification treatments. However, the lowest values (0.44–0.73) were observed for biodiesel samples produced from WFO treated with different levels of adsorbents. These values reveal that treated samples are more resistant to oxidation than those without purification treatment. In almost all the biodiesels, significant amounts of esters of oleic, linoleic, or linolenic acids are present, and the trend of increasing stability was linolenic < linoleic < oleic [47]. These esters undergo auto-oxidation with different rates, depending upon the number and position of the double bonds, resulting in the formation of a series of by-products like acids, esters, aldehydes, ketones, lactones, etc. This renders biodiesel useless as a fuel due to problems experienced during engine operation like fuel filter clogging, fuel atomization, etc. The presence of the above byproducts of auto-oxidation and other reactions affect biodiesel quality and, hence, make it unstable and unfit for use in engines [48]. According to Knothe *et al* [49], high CNs were observed for esters of saturated fatty acids such as palmitic (C16:0) and stearic (C18:0) acids. Palm biodiesel, rich in these compounds, gave the highest CN. Similar findings were reported by Ramos *et al* [46], observing an increase of the CN with increasing the percentage of methyl palmitate in a blend.

**Table 1.** Fatty acid composition of biodiesel produced from WFO and WFO treated with various levels of adsorbents.

			DPSC			SCBA			RHA		
Fatty acid (%)	Control	Magnesol XL 2%	1%	2%	3%	1%	2%	3%	1%	2%	3%
C 10:0 Capric (decanoic) acid	ND	ND	ND	ND	ND	ND	ND	ND	ND	ND	ND
C 12:0 Lauric (dodecanoic) acid	ND	ND	ND	ND	ND	ND	ND	ND	ND	ND	ND
C 14:0 Myristic (tetradecanoic) acid	1.08	1.14	1.00	0.82	0.63	0.96	1.32	0.91	0.67	1.02	1.15
C 16:0 Palmitic (hexadecanoic) acid	44.92	45.90	45.50	46.91	48.94	45.55	43.51	43.82	50.72	49.49	45.49
C 16:1 Palmitoleic (cis-9-hexadecenoic) acid	0.14	0.15	0.93	0.08	ND	0.14	0.36	0.16	0.64	0.05	0.18
C 17:0 margaric (heptadecanoic) acid	ND	ND	ND	0.01	0.55	ND	0.02	0.02	0.09	ND	ND
C 18:0 Stearic (octadecanoic) acid	4.69	4.96	4.30	4.19	4.27	4.57	5.80	4.99	4.34	4.45	4.68
C 18:1 Oleic (cis-9-octadecenoic) acid	40.41	48.13	47.05	47.99	45.62	48.78	48.99	50.1	43.54	44.99	48.5
C 18:2 Linoleic (9,12-octadecadienoic) acid	8.77	ND	ND	ND	ND	ND	ND	ND	ND	ND	ND
C 18:3 Linolenic (9,12,15-octadecatrienoic) acid	ND	ND	1.21	ND	ND	ND	ND	ND	ND	ND	ND
C 20:0 Arachidic (eicosanoic) acid	ND	ND	ND	ND	ND	ND	ND	ND	ND	ND	ND
Saturated fatty acids	50.69	51.73	50.8	51.93	54.39	51.08	50.65	49.74	55.82	54.96	51.32
Monounsaturated fatty acids	40.55	48.28	47.95	48.07	45.62	48.92	49.35	50.26	44.18	45.04	48.68
Polyunsaturated fatty acids	8.77	—	1.21	—	—	—	—	—	—	—	—
COX^a^	1.30	0.48	0.73	0.48	0.45	0.48	0.49	0.50	0.44	0.45	0.48

a COX refers to calculated oxidisability value as reported by Fatemi and Hammond [23].

ND—not detected.

## 4. Conclusions

Purification treatments with various levels of adsorbents caused significant (*p* ≤ 0.05) decreases in FFAs, PVs, and IVs, and no obvious change in SV compared with non-treated oils. The efficiency of the adsorbents under study in improving the physical and chemical properties of biodiesel samples produced from WFO was nearly the same. The highest yields (86.45 and 87.80%) were observed for biodiesel samples produced from WFO treated with 2% Magnesol and 3% of RHA, respectively, followed by samples treated with 2 and 3% of DPSC or RHA. Pre-treatments caused a marked decrease in the content of C 18:2 linoleic acids, consistent with a marked increase in the content of monounsaturated and saturated fatty acids (MUFA) in the treated samples. The highest COX value (1.30) was observed for biodiesel samples produced from WFO without purification treatments. However, the lowest values (0.44–0.73) were observed for biodiesel samples produced from WFO treated with different levels of adsorbent. These values reveal that treated samples are more resistant to oxidation than those without purification treatment.
